# Prevalence of cytopenia and its associated factors among HIV infected adults on highly active antiretroviral therapy at Mehal Meda Hospital, North Shewa Zone, Ethiopia

**DOI:** 10.1371/journal.pone.0239215

**Published:** 2020-09-15

**Authors:** Angesom Gebreweld, Temesgen Fiseha, Nibret Girma, Haftay Haileslasie, Daniel Gebretsadik

**Affiliations:** 1 Department of Medical Laboratory Sciences, College of Health Sciences, Mekelle University, Mekelle, Ethiopia; 2 Department of Medical Laboratory Sciences, College of Medicine and Health Science, Wollo University, Dessie, Ethiopia; 3 Department of Laboratory, Mehal Meda Hospital, Mehal Meda, Ethiopia; University of KwaZulu-Natal School of Social Sciences, SOUTH AFRICA

## Abstract

**Background:**

Cytopenias affect the outcomes of highly active anti-retroviral therapy that results in higher morbidity, mortality, and impaired quality of life. The purpose of this study was to assess the prevalence of cytopenia and its associated factors among HIV infected adults on highly active antiretroviral therapy at Mehal Meda Hospital, North Shewa Zone, Ethiopia.

**Method:**

A cross-sectional health facility based study was conducted among 499 consecutively selected adult HIV infected patients taking HAART for at least six months from January to April 2018. The study participant’s socio-demographic and clinical information was collected using a pre-tested questionnaire and reviewing of medical records by trained clinical nurses. Complete blood count and CD4 T cell count were determined by Sysmex KX-21 N and BD FACS count respectively. Bivariate and multivariate analysis was performed to identify the independently associated factors of cytopenia and prevalence ratios and their 95% confidence intervals were estimated using Poisson regression model with robust error variance to quantify the strength of statistical association. In all cases, a P value less than 0.05 was considered statistically significant.

**Result:**

Out of the total study participants, 39.9% had at least one form of cytopenia, 23.2% had anemia, 13.8% had leukopenia, 12.4% had thrombocytopenia, 11.62% had bi-cytopenias, and only 1% had pancytopenia. In multivariate analysis, cytopenia was independently associated with older age groups, male gender, ZDV based regimen, and CD4 count less than 200 cells/mm^3^.

**Conclusions:**

In this study, the magnitude of any cytopenia was 40% among adult HIV infected patients taking highly active antiretroviral therapy and the prevalence increased as the CD4 count decreases. Therefore, these warrant the need for monitoring hematological parameters of HIV infected patients on HAART to reduce morbidity and mortality.

## Introduction

Human immunodeficiency virus (HIV) continues to be a major global public health issue; 37.9 million people globally were living with HIV and 770,000 people died from acquired immunodeficiency syndrome (AIDS) related illnesses at the end of 2018. Of all people living with HIV, over two-thirds live in Africa [1]. In Ethiopia, an estimated 613,000 people were living with HIV in 2017, 74% of the peoples living with HIV are from Amhara, Oromia, and Addis Ababa [2].

HIV not only targets the immune system that leads to progressive immune dysfunction but also affects the hematopoietic system of the infected individuals that result in cytopenias [3]. Cytopenias are the most common hematological complications of HIV infection and may affect any of the major blood lineages leading to anemia, thrombocytopenia, and/or leucopenia. The causes of cytopenias in HIV infection are multifactorial, including a direct consequence of HIV infection, effects of medications, opportunistic infections, hepatitis B virus and hepatitis C virus co-infection, and others. The pathophysiology of cytopenias can broadly be classified as a bone marrow production defect and increased peripheral loss or destruction of blood cells [3–5]. The frequency and severity of cytopenias increase as CD4 count declines and HIV infection advances. Cytopenias can affect the outcomes of highly active antiretroviral therapy (HAART), resulting in higher morbidity, mortality, and a negative impact on the quality of life [6–8]. Anemia is the most frequent cytopenia in HIV infected patients, even in patients taking HAART. It has been independently associated with accelerated HIV disease progression, mortality and decreased quality of life [9–11]. In HIV patients, the prevalence of anemia varies significantly among studies ranging from 1.3% to 95% [9]. Although treatment with HAART decreases the prevalence of severe anemia [12], the overall burden of anemia in HAART treated HIV patients remains high. In China, anemia occurs in 39.2% HIV patients receiving HAART [13]; in Europe and North America the prevalence is 26% [14]. In Ethiopia, the prevalence of anemia among HIV infected adult individuals receiving HAART ranges from 11.4% to 43% [12, 15–19].

Leucopenia is one of the hematological abnormalities encountered commonly in patients with HIV infection. Neutropenia is the most common leucopenia that occurs in 5–30% of patients with early symptomatic HIV infection and up to 70% of patients at advanced stages of AIDS [8, 20, 21]. Like other cytopenias, the cause of HIV-associated neutropenia is multifactorial, including a direct consequence of HIV infection, autoimmune disorders, opportunistic infections, malignancies, and drugs used to treat HIV and opportunistic infections like antiretroviral therapy (particularly zidovudine-containing regimens), cotrimoxazole, and others, which are myelotoxic [20, 22, 23].

Thrombocytopenia is also commonly observed cytopenia in HIV-infected patients. It may occur in 4.1–40% of HIV patients and the prevalence and severity of thrombocytopenia increases as the disease stage advances [3, 20]. The causes of thrombocytopenia include immune-mediated platelet destruction, impaired megakaryocytosis/direct infection of megakaryocytes, hypersplenism, opportunistic infections, malignancy, and myelosuppression effects of medications [20, 24].

Cytopenia remains problematic in resource limited countries like Ethiopia. The diagnosis of cytopenia and its underlying mechanisms during antiretroviral therapy ensure optimal management of patients with HIV disease [25]. However, in our setting, there is scarce information on the magnitude and factors associated with cytopenias in patients under HAART. Therefore, the purpose of this study was to assess prevalence of cytopenia and its associated factors among HIV infected patients on HAART at Mehal Meda Hospital, North Shewa Zone, Ethiopia.

## Materials and methods

### Study design, setting and population

A cross-sectional study was conducted to assess the prevalence of cytopenia and its associated factors among HIV infected patients on HAART at Mehal Meda Hospital from January to April 2018. The Hospital is found in Mehal Meda town in Menz Gera Midir Woreda North Shewa Zone of the Amhara Region, Ethiopia. Mehal Meda town is about 280 km away from the capital city Addis Ababa and 148 km from Debre Brhan city. Mehal Meda Hospital provides comprehensive health care services including HIV/AIDS diagnosis, treatment and monitoring for a catchment population of more than 360,000.

A total of 499 consecutively selected adult HIV infected patients taking HAART for at least six months were enrolled from ART follow-up unit of Mehal Meda Hospital. The sample size was calculated using single proportion formula with an assumption of a proportion of cytopenia among HIV infected patients taking HAART of 50% since there is no study conducted in the area. The assumptions also include 95% confidence interval, 5% marginal error and 10% non-response rate. The calculated sample size become 422 but we included 499 subjects into the study. HIV-infected patients with known hematological disorders, pregnancy, postpartum period, blood transfusion (within three months) and patients younger than 18 years were excluded from the study.

### Data collection procedure

The study participant’s socio-demographic and clinical information was collected using a pre-tested questionnaire and by reviewing of medical records by trained clinical nurses. Height and weight of the participants were measured by the data collectors.

Venous blood specimen (about 4 ml) in EDTA vacutainer tube was collected from each study participants by a senior laboratory professional for complete blood count and CD4 T cell count determination. Sysmex KX-21N (Sysmex corporation kobe, Japan) automated hematological analyzer were used to determine complete blood count (total white blood cell count (WBC), red blood cell count, platelet (PLT) count, and hemoglobin concentration level) and BD FACS count (Becton Dickenson and Company, California, USA) machine was used to measure CD4 T cell count of the study participants. Standard operating procedures and manufacturers’ instructions were strictly followed in each laboratory procedures to maintain quality of the laboratory results. Expiry date of reagents were checked and quality control materials were run along with patient samples to check precision of the instruments and accuracy of the results.

### Operational definitions

Anemia was defined based on the World Health Organization (WHO) criteria: hemoglobin (Hgb) concentration <13 g/dl for males (15 years of age and above) and < 12 g/dl for females (non-pregnant women). Anemia was also categorized using the WHO classification into mild anemia (for men: Hgb level from 11.0–12.9 g/dl, for non-pregnant women: Hgb level from 11.0–11.9 g/dl), moderate anemia (Hgb level from 8.0–10.9 g/dl for both sexes), and severe anemia (Hgb lower than 8.0 g/dl for both sexes) [26].

As anemia, there is no generally accepted cut-offs for leucopenia and thrombocytopenia; we defined them as used in other studies [19, 27]. Leucopenia was defined as a total WBC < 4.0 × 103/μL, whereas thrombocytopenia was defined as a total platelet count < 150 × 103/μL. The platelet counts from 100 to150 × 103/μL, 50 to 100 × 103/μL, and less than 50 × 103/μL were considered as mild thrombocytopenia, moderate thrombocytopenia, and severe thrombocytopenia, respectively [28].

Any cytopenia was defined as presence of at least one form of cytopenia (anemia, thrombocytopenia, or leucopenia), bi-cytopenia was defined as presence of two forms of cytopenias (anemia, thrombocytopenia and leucopenia) and pancytopenia was defined as having all three forms of cytopenia simultaneously on the study participants.

### Statistical analysis

All collected data were entered in to “Epi Data version 3.1” and exported to Stata® 12.0 (StataCorp, College Station, Texas) statistical software for analysis. Continuous variables were expressed as median and inter-quartile range (IQR) and categorical variables were reported as frequencies and percentages. Chi-square (×2) test for categorical variables was performed to assess their relation with the outcomes. Bivariate and multivariate analysis was performed to identify the independently associated factors of cytopenias and prevalence ratios and their 95% confidence intervals were estimated using Poisson regression model with robust error variance to quantify the strength of statistical association. Variables with p < 0.20 at bivariate analysis were included in the multivariable analysis. In all cases, a P value less than 0.05 was considered statistically significant.

### Ethical considerations

Ethical approval was obtained after the study protocol was approved by Research and Ethics Review Committee of the Department of Medical Laboratory Sciences, Wollo University and a letter of permission to conduct the study was obtained from Mehal Meda Hospital. Individual written informed consent was obtained after the purpose and importance of the study were explained. To ensure confidentiality, participant’s identifiers were removed and only code numbers were used throughout the study. Any abnormal test results of participants were reported to the concerned body in the ART clinic of the hospital.

## Result

### Sociodemographic characteristics of study participants

A total of 499 HIV positive patients taking ART were included in this study. The median age of the study participants was 36 years (Interquartile Range: 29–43) and most of the study participants (n = 163; 32.7%) were within 30–39 years of age. Of the total 499 study participants, 260 (52.1%) were females, 301 (60.3%) were from urban setting, 394 (79.0%) had primary education and below, 240 (48.1%) were married and 163 (32.7%) were employed (Table 1).

**Table 1:** Sociodemographic characteristics of HIV positive patients taking ART at Mahal Meda Hospital, North Shewa, Ethiopia, 2018 (n = 499).

Variables	Category	Frequency (n = 499)	Percentage (%)
Age (in years)	18–29	138	27.7
	30–39	163	32.7
	40–49	149	29.9
	> = 50	49	9.8
Sex	Male	239	47.9
	Female	260	52.1
Residence	Urban	301	60.3
	Rural	198	39.7
Educational status	Primary school and below	394	79.0
	High school and above	105	21.0
Occupational status	Employed	163	32.7
	Unemployed	336	67.3
Marital status	Married	240	48.1
	Unmarried	259	51.9
Family size	Three and below	273	54.7
	Above three	226	45.3

### Clinical and laboratory characteristics of the study participants

Among the study participants, 295 (59.1%) were in WHO clinical stage I and 226 (45.3%) were taking 1e (TDF-3TC-EFV) ART regimen. Majority of the study participants (n = 329; 65.9%) were used the ART regimen with in duration group of 12–59 months with median of 31.0 months (interquartile range: 17.0–60.0 months). One hundred twenty nine (25.9%) of the study participants had opportunistic infection (tuberculosis; 24 (4.8%), pneumonia; 34 (6.8%), oral candidiasis; 7 (1.4%), and diarrhea; 64 (12.8%)) and 30 (6%) of the study participants had chronic disease (diabetic mellitus 9 (1.8%) and hypertension 21 (4.2%)). The median body mass index of the study participants was 19.65 Kg/m^2^ (interquartile range: 17.96–21.33 Kg/m2) and 150 (30.1%) study participants were under weight (BMI<18.5 Kg/m^2^). Majority of the respondents 245 (49.1%) had CD4 cell count greater than 500 cell/μL while only 70 (14%) of the respondents had less than 200 cell/μL. The median CD4 count, hemoglobin concentration, white blood cell count and platelet count was 482.0 cell/μL (interquartile range: 290.0–669.0 cell/μL), 14.20 g/dL (interquartile range: 12.60–15.60 g/dL), 6.40 x10^3^/μL (interquartile range: 4.80–7.90 x10^3^/μL), and 242.0 x10^3^/μL (interquartile range: 194.0–301.0 x10^3^/μL), respectively (Table 2).

**Table 2:** Clinical and laboratory characteristics of HIV infected patients on HAART at Mahal-Meda Hospitals, Northern Shewa Zone, Ethiopia, 2018 (n = 499).

Variables	Category	Frequency (n = 499)	Percentage (%)	Median (IQR)
WHO clinical stages	Stage I	295	59.1	-
	Stage II	145	29.1	-
	Stage III	53	10.6	-
	Stage IV	6	1.2	-
Types of ART regimens	1C	163	32.7	-
	1D	89	17.8	-
	1E	226	45.3	-
	1F	10	2.0	-
	1H	11	2.2	-
Duration of ART regiment	<12 months	46	9.2	-
	12–59 months	329	65.9	-
	> = 60 months	124	24.8	-
Opportunistic infection	TB	24	4.8	-
	Pneumonia	34	6.8	-
	Oral candidiasis	7	1.4	-
	Diarrhea	64	12.8	-
	No	370	74.1	-
Chronic illness	Diabetic Mellitus	9	1.8	-
	Hypertension	21	4.2	-
	NO	469	94.0	-
BMI category	18.5–24.9	330	66.1	-
	<18	150	30.1	-
	> = 25	19	3.8	-
CD4 count (cells/μL)	<200	71	14.2	-
	200–499	183	36.7	-
	> = 500	245	49.1	-
Duration in months		-	-	31.0 (17.0–60.0)
BMI Kg/m2		-	-	19.65 (17.96–21.33)
CD4 count (cells/μL)		-	-	482.0 (290.0–669.0)
White blood cell count (x10^3^/μL)		-	-	6.40 (4.80–7.90)
Hemoglobin concentration (g/dL)		-	-	14.20 (12.60–15.60)
Platelet count (x10^3^/μL)		-	-	242.0 (194.0–301.0)

WHO = World Health Organization, IQR = interquartile range, 1c = ZDV -3TC-NVP, 1d = ZDV-3TC-EFV, 1e = TDF-3TC-EFV, 1f = TDF-3TC-NVP, 1h = ABC+3TC+EFV, ZDV = zidovudine, 3TC = lamuvidine, EFV = efaverenz, NVP = neverapine, TDF = tenofovir, ABC = abacavir, BMI = body mass index.

### Prevalence and potential risk factors of cytopenias

Out of the total study participants, 39.9% had any cytopenias, 23.2% had anemia, 13.8% had leukopenia, 12.4% had thrombocytopenia, 11.62% had bi-cytopenias, and only 1% had Pancytopenia. The most frequent bi-cytopenias was anemia and thrombocytopenia combination (4.2%). Of the anemic study subjects (n = 116), 8.62% had severe anemia, 54.31% had moderate anemia, and 37.07% had mild anemia. Majority of the thrombocytopenic study participants had mild thrombocytopenia (87.1%) (Table 3).

**Table 3:** Prevalence of cytopenias among HIV infected patients on HAART at Mahal-Meda Hospitals, Northern Shewa Zone, Ethiopia, 2018 (n = 499).

Hematological abnormalities	Frequency (%)
**Any cytopenia**	199 (39.9)
Anemia	116 (23.2)
Leucopenia	69 (13.8)
Thrombocytopenia	62 (12.4)
**Bi-cytopenia**	
Anemia and leucopenia	18 (3.6)
Anemia and thrombocytopenia	21 (4.2)
Thrombocytopenia and leucopenia	14 (2.8)
**Pancytopenia**	5 (1.0)
**Anemia severity (n = 116)**	
Mild	43 (37.07)
Moderate	63 (54.31)
Severe	10 (8.62)
**Thrombocytopenia severity (n = 62)**	
Mild	54 (87.10)
Moderate	4 (6.45)
Severe	4 (6.45)

Mild anemia = (Hgb level: 11.0–12.9 g/dl for men, 11.0–11.9 g/dl for women), moderate anemia (Hgb level 8.0–10.9 g/dl for both sexes), severe anemia (Hgb level <8.0 g/dl for both sexes), mild thrombocytopenia (platelet counts: 100 to150 × 103/μL), moderate thrombocytopenia (platelet counts: 50 to 100 × 103/μL), severe thrombocytopenia (< 50 × 103/μL).

The prevalence of any cytopenia (at least one form of cytopenia) was significantly associated with male patients, older age groups, rural dwellers, being unmarried by marital status, WHO clinical stage II, ZDV based regimen, taking ART regimen below 60 months, presence of opportunistic infection, and CD4 count less than 200 cells/mm^3^ and 200–499 cells/mm^3^on the bivariate analysis.

Using multivariate logistic regression analysis, cytopenia was independently associated with male gender (PR = 1.34, 95% CI: 1.07–1.67, P = .011), unmarried (PR = 1.36, 95% CI: 1.09–1.69, P = 0.007), ZDV based regimen (PR = 1.56, 95% CI: 1.24–1.96, P<0.001), taking ART regiment for 12–60 months (PR = 1.54, 95% CI: 1.16–2.06, P = 0.003), CD4 count less than 200 cells/mm^3^ (PR = 2.06, 95% CI: 1.54–2.75, P<0.001) and 200–499 cells/mm^3^ (PR = 1.36, 95% CI: 1.06–1.74, P = 0.015) (Table 4).

**Table 4:** Factors associated with cytopenia among HIV infected patients on HAART at Mahal-Meda Hospitals, Northern Shewa Zone, Ethiopia, 2018 (n = 499).

Variable	Any Cytopenia		Bivariable	P-value	Multivariable	P-value
	No, n (%)	Yes, n (%)	PR (95% CI)		PR (95% CI)	
**Age group (in years)**						
18–29	83 (60.1)	55 (39.9)	1		1	
30–39	103 (63.2)	60 (36.8)	0.92 (0.69–1.23)	0.587	1.02 (0.75–1.37)	0.918
40–49	94 (63.1)	55 (36.9)	0.93 (0.69–1.24)	0.608	0.93 (0.68–1.28)	0.667
> = 50	20 (40.8)	29 (59.2)	**1.48 (1.09–2.03)**	**0.012**	**1.33 (0.93–1.90)**	0.116
**Sex**						
Male	124 (51.9)	115 (48.1)	**1.49 (1.19–1.86)**	**<0.001**	**1.34 (1.07–1.67**)	0.011
Female	176 (67.7)	84 (32.3)	1		1	
**Residence**						
Urban	193 (64.1)	108 (35.9)	1		1	
Rural	107 (54.0)	91 (46.0)	**1.28 (1.04–1.59)**	**0.023**	1.22 (0.98–1.52)	0.076
**Educational status**						
Primary school and below	242 (61.4)	152 (38.6)	0.86 (0.67–1.10)	0.237		
High school and above	58 (55.2)	47 (44.8)	1			
**Occupational status**						
Employed	105 (64.4)	58 (35.6)	1		1	
Un employed	195 (58.0)	141 (42.0)	1.18 (0.93–1.50)	0.181	1.01 (0.80–1.28)	0.914
**Marital status**						
Married	164 (68.3)	76 (31.7)	1		1	
Unmarried	136 (52.5)	123 (47.5)	**1.50 (1.19–1.88)**	<0.001	**1.36 (1.09–1.69)**	0.007
**WHO clinical stage**						
stage I	191 (64.7)	104 (35.3)	1		1	
Stage II	77 (53.1)	68 (46.9)	**1.33 (1.05–1.68)**	**0.016**	1.13 (0.90–1.41)	0.295
Stage III and IV	32 (54.2)	27 (45.8)	1.29 (0.95–1.78)	0.108	0.81 (0.58–1.13)	0.221
**Regimen**						
ZDV based	123 (48.8)	129 (51.2)	**1.81 (1.43–2.28)**	<0.001	**1.55 (1.24–1.96)**	<0.001
Non ZDV based	177 (71.7)	70 (28.3)	1		1	
**Duration**						
<12 months	24 (52.2)	22 (47.8)	**1.60 (1.07–2.40)**	0.022	1.49 (0.97–2.32)	0.071
12–60 months	189 (57.4)	140 (42.6)	**1.43 (1.06–1.92)**	0.019	**1.54 (1.16–2.06)**	0.003
>60 months	87 (70.2)	37 (29.8)	1		1	
**Opportunistic infection**						
Yes	59 (45.7)	70 (54.3)	**1.56 (1.26–1.92)**	**<0.001**	1.05 (0.84–1.32)	0.655
No	241 (65.1)	129 (34.9)	1		1	
**Chronic disease**						
Yes	14 (46.7)	16 (53.3)	1.37 (0.96–1.95)	0.083	0.97 (0.65–1.44)	0.878
No	286 (61.0)	183 (39.0)	1		1	
**BMI category**						
18.5–24.9	198 (59.8)	133 (40.2)	1			
<18	88 (59.1)	61 (40.9)	1.02 (0.81–1.29)	0.875		
> = 25	14 (73.7)	5 (26.3)	0.65 (0.31–1.41)	0.277		
**CD4 count category**						
<200	26 (36.6)	45 (63.4)	**2.19 (1.68–2.85)**	<0.001	**2.06 (1.54–2.75)**	<0.001
200–499	100 (54.6)	83 (45.4)	**1.56 (1.22–2.01)**	0.001	**1.36 (1.06–1.74)**	0.015
> = 500	174 (71.0)	71 (29.0)	1		1	

WHO = World Health Organization, ZDV = zidovudine, PR = Prevalence ratio, CI = Confidence interval, 1.00 = reference group.

The prevalence of anemia was significantly higher in males than females (28% vs18.8%, P = 0.016). Anemia was also higher in HIV patients with older age group, rural residents, WHO clinical stage III and IV, ZDV based ART regimen, and opportunistic infection. The prevalence of anemia was decreased as ART regiment usage becomes longer. However, the prevalence of anemia increased as the CD4 T cell count decreased (Table 5).

**Table 5:** Factors associated with anemia among HIV infected patients on HAART at Mahal-Meda Hospitals, Northern Shewa Zone, Ethiopia, 2018 (n = 499).

Variable	Anemia		Bivariable	P-value		Multivariable	P-value
	No, n (%)	Yes, n (%)	PR (95% CI)			PR (95% CI)	
Age group (in years)							
18–29	106 (76.8)	32 (23.2)	1			1	
30–39	126 (77.3)	37 (22.7)	0.98 (0.65–1.48)	0.920		0.98 (0.62–1.55)	0.926
40–49	121 (81.2)	28 (18.8)	0.81 (0.52–1.27)	0.362		0.84 (0.52–1.35)	0.463
> = 50	30 (61.2)	19 (38.8)	**1.68 (1.05–2.66)**	**0.030**		**1.39 (1.05–2.32)**	0.027
**Sex**							
Male	172 (72.0)	67 (28.0)	**1.49 (1.08–2.06)**	**0.016**		1.22 (0.89–1.67)	0.210
Female	211 (81.2)	49 (18.8)	1			1	
**Residence**							
Urban	253 (84.1)	48 (15.9)	1			1	
Rural	130 (65.7)	68 (34.3)	**2.15 (1.56–2.98)**	**<0.001**		**2.05 (1.48–2.84)**	<0.001
**Educational status**							
Primary school and below	305 (77.4)	89 (22.6)	0.88 (0.60–1.28)	0.496			
High school and above	78 (74.3)	27 (25.7)	1				
**Occupational status**							
Employed	131 (80.4)	32 (19.6)	1			1	
Un employed	252 (75.0)	84 (25.0)	1.27 (0.89–1.83)	0.191		0.88 (0.61–1.28)	0.516
**Marital Status**							
Married	199 (82.9)	41 (17.1)	1			1	
Unmarried	184 (71.0)	75 (29.0)	**1.69 (1.21–2.38)**		0.002	**1.46 (1.04–2.06)**	0.028
**WHO clinical stage**							
Stage I	243 (82.4)	52 (17.6)	1			1	
Stage II	100 (69.0)	45 (31.0)	**1.76 (1.25–2.50)**		**0.001**	**1.51 (1.08–2.10)**	0.016
Stage III and IV	40 (67.8)	19 (32.2)	**1.83 (1.17–2.85)**		**0.008**	1.12 (0.69–1.79)	0.649
**Regimen**							
ZDV based	178 (70.6)	74 (29.4)	**1.73 (1.24–2.42)**		0.001	1.25 (0.89–1.74)	0.187
Non ZDV based	205 (83.0)	42 (17.0)	1			1	
**Duration**							
<12 months	33 (71.7)	13 (28.3)	**2.08 (1.09–3.90)**		**0.026**	**2.03 (1.01–4.07)**	0.046
12–60 months	243 (73.9)	86 (26.1)	**1.91 (1.18–3.07)**		**0.008**	**1.93 (1.24–3.02)**	0.004
>60 months	107 (86.3)	17 (13.7)	1			1	
**Opportunistic infection**							
Yes	76 (58.9)	53 (41.1)	**2.41 (1.78–3.28)**		**<0.001**	**1.69 (1.13–2.23)**	0.007
No	307 (83.0)	63 (17.0)	1			1	
**Chronic disease**							
Yes	19 (63.3)	11 (36.7)	1.64 (0.99–2.70)		0.053	1.05 (0.59–1.88)	0.859
No	364 (77.6)	105 (22.4)	1			1	
**BMI category**							
18.5–24.9	258 (77.9)	73 (22.1)	1				
<18	111 (74.5)	38 (25.5)	1.16 (0.82–1.63)	0.404			
> = 25	14 (73.7)	5 (26.3)	1.19 (0.54–2.61)	0.657			
**CD4 count category**							
<200	47 (66.2)	24 (33.8)	**1.76 (1.16–2.67)**	**0.007**		**1.53 (1.01–2.31)**	0.045
200–499	138 (75.4)	45 (24.6)	1.28 (0.89–1.84)	0.178		0.91 (0.65–1.28)	0.594
> = 500	198 (80.8)	47 (19.2)	1			1	

WHO = World Health Organization, ZDV = Zidovudine, PR = Prevalence ratio, CI = Confidence interval, 1.00 = Reference group.

In multivariable analysis, the prevalence ratio of having anemia in older age group (≥50 years) was 1.4 (PR = 1.39, 95% CI: 1.05–2.32, P = 0.027) compared to younger age group (18–29 years). The prevalence ratio of anemia was twice in rural residents (PR = 2.05, 95% CI: 1.48–2.84, P<0.001) compared to urban residents, 1.5 (PR = 1.46, 95% CI: 1.04–2.06, P = 0.028) in unmarried study participants compared to married participants, and 1.5 times higher in WHO clinical stage II (PR = 1.51, 95% CI: 1.08–2.10, P = 0.016) compare to participants in WHO clinical stage I. Anemia was also independently associated with 12–60 months duration of ART regimen (PR = 1.93, 95% CI: 1.24–3.02, P = 0.004) and opportunistic infection (PR = 1.69, 95%CI: 1.13–2.23, P = 0.007) as summarized in Table 5.

The prevalence of leucopenia among males and females were 13.4% and 14.2%, respectively but the difference were not statistically significant (P = 0.786). Leucopenia prevalence progressively increased with age: 8.0% for age 18–29 years, 10.4% for 30–39 years, 18.8% for 40–49 years and 26.5% for >50 years (P = 0.002). Leucopenia had decreased as CD4+ T-cell counts increased: CD4 count <200 cells/mm^3^ (38.0%), CD4 count from 200–499 cells/mm^3^ (15.8%), and CD4 count >500 cell/mm^3^ (5.3%) (P<0.001). Using a multivariate analysis, leucopenia was independently associated with age category 40–49 years (PR = 2.06, 95% CI: 1.12–3.79, P = 0.019), >50 years (PR = 2.40, 95% CI: 1.26–4.61, P = 0.008), ZDV based regimen (PR = 1.86, 95% CI: 1.15–3.00, P = 0.004), CD4 count less than 200 cells/mm^3^ (PR = 5.99, 95% CI: 3.18–11.29, P<0.001), and CD4 count from 200–499 cells/mm^3^ (PR = 2.91, 95% CI: 1.56–5.42, P = 0.001) (Table 6).

**Table 6:** Factors associated with leucopenia among HIV infected patients on HAART at Mahal-Meda Hospital, Northern Shewa Zone, Ethiopia, 2018 (n = 499).

Variable	Leucopenia		Bivariable	P value	Multivariable	P-value
	No, n (%)	Yes, n (%)	PR (95% CI)		PR (95% CI)	
**Age group (in years)**						
18–29	127 (92.0)	11 (8.0)	1		1	
30–39	146 (89.6)	17 (10.4)	1.31 (.63–2.70)	0.467	1.61 (0.81–3.19)	0.172
40–49	121 (81.2)	28 (18.8)	**2.36 (1.22–4.55)**	**0.011**	**2.06 (1.12–3.79)**	0.019
> = 50	36 (73.5)	13 (26.5)	**3.33 (1.59–6.94)**	**0.001**	**2.40 (1.26–4.61)**	0.008
**Sex**						
Male	207 (86.6)	32 (13.4)	**1**			
Female	223 (85,8)	37 (14.2)	1.06 (0.68–1.65)	0.786		
**Residence**						
Urban	257 (85.4)	44 (14.6)	1			
Rural	173 (87.4)	25 (12.6)	0.86 (0.55–1.36)	0.530		
**Educational status**						
Primary school and below	344 (87.3)	50 (12.7)	0.70 (0.43–1.14)	0.150	0.70 (0.44–1.12)	0.142
High school and above	86 (81.9)	19 (18.1)	1		1	
**Occupational status**						
Employed	138 (84.7)	25 (15.3)	1			
Un employed	292 (86.9)	44 (13.1)	0.85 (0.54–1.35)	0.495		
**Marital status**						
Married	205 (85.4)	35 (14.6)	1			
Unmarried	225 (86.9)	34 (13.1)	0.90 (0.58–1.40)	0.638		
**WHO clinical stage**						
stage I	254 (86.1)	41 (13.9)	1		1	
Stage 2	133 (91.7)	12 (8.3)	0.59 (0.32–1.09)	0.097	**0.42 (0.23–0.76)**	0.004
Stage 3and 4	43 (72.9)	16 (27.1)	**1.95 (1.18–3.23)**	**0.010**	1.34 (0.78–2.29)	0.727
**Regimen**						
ZDV based	206 (81.7)	46 (18.3)	**1.96 (1.23–3.13)**	0.005	**1.86 (1.15–3.00)**	**0.011**
Non ZDV based	224 (90.7)	23 (9.3)	1			
**Duration**						
<12 months	38 (82.6)	8 (17.4)	**1**			
12–60 months	290 (88.1)	39 (11.9)	0.681 (0.34–1.37)	0.280		
>60 months	102 (82.3)	22 (17.7)	1.02 (0.49–2.13)	0.958		
**Opportunistic infection**						
Yes	113 (87.6)	16 (12.4)	0.86 (0.51–1.46)	0.589		
No	317 (85.7)	53 (14.3)	1			
**Chronic disease**						
Yes	26 (86.7)	4 (13.3)	0.96 (0.38–2.46)	0.936		
NO	404 (86.1)	65 (13.9)	1			
**BMI category**						
18.5–24.9	281 (84.9)	50 (15.1)	1			
<18	130 (87.2)	19 (12.8)	0.84 (0.52–1.38)	0.500		
> = 25	19 (100)	0 (0)	0.00 (0.00)	0.985		
**CD4 count category**						
<200	44 (62.0)	27 (38.0)	**7.16 (3.91–13.15)**	**<0.001**	**5.99 (3.18–11.29)**	**<0.001**
200–499	154 (84.2)	29 (15.8)	**2.99 (1.59–5.59)**	**0.001**	**2.91 (1.56–5.42)**	**<0.001**
> = 500	232 (94.7)	13 (5.3)	1		1	

WHO = World Health Organization, ZDV = Zidovudine, PR = Prevalence ratio, CI = Confidence interval, 1.00 = Reference group.

In this study, the overall prevalence of thrombocytopenia was 12.4%. The prevalence of thrombocytopenia among males (17.6%) were higher than females (7.7%) and the difference were statistically significant (P = .001). In bivariate analysis, thrombocytopenia was associated with age group, male gender, occupational status, marital status, WHO clinical stage III and IV, AZT based ART regimen, CD4 count less than 200 cell/mm^3^. In multivariate analysis, thrombocytopenia was independently associated with age group 40–49 years (PR = 0.33, 95% CI: 0.16–0.70, P = 0.004), male participants (PR = 2.36, 95% CI: 1.37–4.07, P = 0.002), unmarried (PR = 2.36, 95% CI: 1.33–4.20, P = 0.003), and CD4 count less than 200 cell/mm^3^ (PR = 2.91, 95% CI: 1.58–5.35, P = 0.001) (Table 7).

**Table 7:** Factors associated with thrombocytopenia among HIV infected patients on HAART at Mahal-Meda Hospitals, Northern Shewa Zone, Ethiopia, 2018 (n = 499).

Variable	Thrombocytopenia		Bi-variable	P value	Multivariable	P-value
	No, n (%)	Yes, n (%)	PR (95% CI)		PR (95% CI)	
**Age group (in years)**						
18–29	112 (81.2)	26 (18.8)	1		1	
30–39	147 (90.2)	16 (9.8)	0.52 (0.29-.93)	0.028	**0. 69 (0.38–1.23)**	0.208
40–49	140 (94.0)	9 (6.0)	**0.32 (0.16–0.66)**	**0.002**	**0.33 (0.16-.70)**	0.004
> = 50	38 (77.6)	11 (22.4)	1.19 (0.64–2.23)	0.583	0.91 (0.45–1.84)	0.801
Sex						
Male	197 (82.4)	42 (17.6)	**2.28 (1.38–3.78)**	**0.001**	**2.36 (1.37–4.07)**	**.002**
Female	240 (92.3)	20 (7.7)	1		1	
**Residence**						
Urban	260 (86.4)	41 (13.6)	1			
Rural	177 (89.4)	21 (10.6)	0.78 (0.47–1.28)	0.322		
**Educational status**						
Primary school and below	352 (89.3)	42 (10.7)	0.56 (0.34–0.91)	0.020	0.82 (0.54–1.25)	0.356
High school and above	85 (81.0)	20 (19.0)	1		1	
Occupational status						
Employed	151 (92.6)	12 (7.4)	1		1	
Un employed	286 (85.1)	50 (14.9)	**2.02 (1.11–3.69)**	0.022	1.57 (0.85–2.88)	0.147
Marital status						
Married	226 (94.2)	14 (5.8)	1		1	
Unmarried	211 (81.5)	48 (18.5)	**3.18 (1.79–5.61)**	<0.001	**2.36 (1.33–4.21)**	0.003
**WHO clinical stage**						
Stage I	265 (89.8)	30 (10.2)	1		1	
Stage 2	125 (86.2)	20 (13.8)	1.36 (0.79–2.31)	0.260	1.15 (0.68–1.96)	0.595
Stage 3and 4	47 (79.7)	12 (20.3)	**2.00 (1.09–3.68)**	**0.026**	1.17 (0.62–2.21)	0.624
**Regimen**						
ZDV based	210 (83.3)	42 (16.7)	2.06 (1.24–3.40)	0.005	1.40 (0.82–2.33)	0.227
Non ZDV based	227 (91.9)	20 (8.1)	1		1	
**Duration**						
<12 months	43 (93.5)	3 (6.5)	1			
12–60 months	285 (86.6)	44 (13.4)	2.05 (.67–6.34)	0.213		
>60 months	109 (87.9)	15 (12.1)	1.86 (0.56–6.11)	0.310		
**Opportunistic infection**						
Yes	109 (84.5)	20 (15.5)	1.366 (0.83–2.24)	0.216		
No	328 (88.6)	42 (11.4)	1			
**Chronic disease**						
Yes	23 (76.7)	7 (23.3)	1.99 (0.99–3.99)	0.052	1.28 (0.60–2.74)	0.519
NO	414 (88.3)	55 (11.7)	1		1	
**BMI category**						
18.5–24.9	291 (87.9)	40 (12.1)	1			
<18	128 (85.9)	21 (14.1)	1.17 (0.71–1.91)	0.540		
> = 25	18 (94.7)	1 (5.3)	0.43 (0.06–3.01)	0.399		
**CD4 count category**						
<200	55 (77.5)	16 (22.5)	**2.40 (1.342–4.29)**	**0.003**	**2.91 (1.58–5.35)**	**0.001**
200–499	160 (87.4)	23 (12.6)	1.34 (0.77–2.31)	0.295	1.12 (0.64–1.98)	0.684
> = 500	222 (90.6)	23 (9.4)	1		1	

WHO = World Health Organization, ZDV = zidovudine, BMI = body mass index, PR = Prevalence ratio, CI = Confidence interval, 1.00 = reference group.

## Discussion

This study assessed the prevalence and associated factors of cytopenias among adult HIV infected patients on HAART at Mehal Meda Hospital, North Shewa Zone, Ethiopia. The overall prevalence of at least one form of cytopenia (presence of anemia, thrombocytopenia or leukopenia) was 39.9% (95% CI;35.5% - 43.5%) and it was independently associated with older age groups, male gender, unmarried marital status, ZDV based ART regimen, taking ART regiment for 12–59 months, and CD4 count less than 200 cells/mm^3^. Prevalence of cytopenia in this study was higher than study done in Beijing Ditan Hospital, China [29]. These differences could be due to different cut-off values used to define the cytopenias, study population, socioeconomic status and dietary habits of study participants.

In this study, the most frequent type of cytopenia were anemia and its prevalence was 23.2% (95% CI; 19.4% - 26.8%) which was in agreement with other findings reported in Gondar (22.2%) [17], Northeastern Nigeria (24.3%) [30], and Kaduna State, Nigeria (23%) [31]. However, the prevalence was lower compared to studies conducted in Debre Tabor (29.9%) [16], Tikur Anbessa Specialized Hospital, Addis Ababa (34.6%) [18], Jimma University Specialized Hospital, Jimma (43.1) [19], South West Region of Cameroon (58.6%) [32], Benin City, Nigeria (51.15%) [33], and Brazil (37.5%) [34]. Our finding was higher than studies conducted in Gondar University Hospital (11.7%) [15], Black Lion Specialized Hospital, (11.4%) [12], and Zewditu Memorial Hospital (14.3%) [35]. The reasons for the observed differences in prevalence of anemia might be due to the difference in study population, socioeconomic status and dietary habits of study participants, sample size, and difference in the definition of anemia.

Of the anemic study subjects, about 9% had severe anemia, 54% had moderate anemia, and 37% had mild anemia. The predominance of moderate type of anemia in the current study is in line with a study conducted at Tikur Anbessa Specialized Hospital, Addis Ababa [18] but it deviates from the findings reported in Wolita Sodo University, Sodo [36], Black Lion Hospital, Addis Ababa [12], Debre Tabor [37], and rural China [13], which reported high rate of mild anemia.

In the present study, the prevalence of anemia was significantly higher in males than females which is consistent with other studies [13, 33, 35]; this might be due to the difference in the definition of anemia. However, many studies reported a high prevalence of anemia in females than male HIV infected patients [18, 37–40]. Similar to other studies findings [12, 13, 19, 39], we found that the prevalence of anemia was significantly higher as the CD4 T cell count of the study participants decreased and WHO clinical stage advanced. This might be due to high HIV infection that leads to compromised immune system and disrupts normal hematopoiesis by cytokine dysregulation [3, 4]. Anemia also independently associated with older age group (≥50 years) and opportunistic infections in this study, this might be associated with immunosuppression. The prevalence of anemia was decreased with longer ART regiment usage and this is attributed to the positive effect of ART on the differentiation and survival of erythrocytes. The finding is supported by other studies [12, 32].

Leukopenia was found in 13.8% (95% CI; 11.1% - 17.1%) of the study participants, this finding was supported by a study conducted in Jimma, Ethiopia (12.3%) [19]. However, the prevalence was lower than studies in Gonder, Ethiopia (35.9%) [15], South West Region of Cameroon (20%) [32], and Karnataka, India (35%) [41] and higher than studies in Ghana (6.5%) [42], Kaduna State, Nigeria (9%) [31], and Ranchi, India (3%) [43]. The observed difference in the prevalence might be due to variation in study populations, clinical conditions, study design methods, and leukopenia definition. In the present study, leukopenia were significantly increased as the CD4 T cell count decreased and age of HIV infected patients increased, which is in agreement with other studies [15, 19, 38]. This could be due to HIV mediated hematopoietic inhibition and direct infection of T cells [4].

Our study showed that the overall prevalence of thrombocytopenia was 12.4% (95% CI; 9.6% - 15.4%). It is consistent with other reports conducted in South West Region of Cameroon (14%) [32] and Southwestern Uganda (13%) [44]. The possible causes of thrombocytopenia could be immune-mediated platelet destruction, impaired platelet production by the infected magakaryocytes of the bone marrow, or myelosuppression effects of medications. On the other hand, the prevalence of this study was higher than studies done in northwest Ethiopia (4.1% and 6.3%) [15, 17], Jimma (6.9%) [19], Addis Ababa (5.7%) [45], and Yaoundé, Cameroon (6.9%) [46], and lower than studies in Ghana (18.5%) [42] and Kaduna State, Nigeria (24%) [26, 31]. The difference might be due to variation in the definition of thrombocytopenia, study design and size of the study population. Regarding the severity of thrombocytopenia, the predominant form was mild thrombocytopenia which is consistent with other studies [45, 46].

Similar to our finding, different studies reported thrombocytopenia were increased and independently associated with degree of immunosuppression [15, 31, 45]. However, our finding deviated from a study conducted in Jimma [19] that showed thrombocytopenia is not associated with neither the degree of immunosuppression nor with the clinical stage of HIV.

Pancytopenia in this study was 1% (95% CI: 0.4%-2%), which is nearly in agreement with a study conducted by Firnhaber C et al (0.3%) [47]. However, a study from Jimma reports no pancytopenia found [19] and studies conducted by Bukar A et al [31] and Santiago-Rodríguez EJ et al [48] reported 8% and 8.7% pancytopenia, respectively, which is higher than our finding. The difference might be due to variation in sample size and design of the studies.

The main limitation of this study is the cross-sectional nature of the study design which does not reveal causal links between independent variables and cytopenias, so a longitudinal study is recommended to generalize the related outcomes of this study. Despite the limitations, the study has determined the magnitude of cytopenias and identified important factors associated with cytopenias in HIV patients on HAART.

In conclusion, in this study prevalence of at least one form of cytopenia was 40% among HIV infected patients on HAART. The most frequent type of cytopenias was anemia followed by leukopenia and thrombocytopenia. Older age group (>50 years old), male gender, ZDV based ART regimen, and lower CD4 T cell count were identified as independent factors associated with having cytopenias (anemia, leucopenia or thrombocytopenia). Therefore, these warrant the need for monitoring hematological parameters of HIV infected patients on HAART to reduce morbidity and mortality.

## Supporting information

S1 Dataset (XLSX)

## References

[1] UNAIDS. Global HIV & AIDS statistics, 2019 fact sheet. https://www.unaids.org/en/resources/fact-sheet. Accessed 20 January 2020.

[2] FHAPCO. HIV Prevention in Ethiopia National Road Map 2018–2020. 2018. https://ethiopia.unfpa.org/sites/default/files/pub-pdf/HIV%20Prevention%20in%20Ethiopia%20National%20Road%20Map%202018%20-%202020%20FINAL_FINAL.pdf. Accessed 15 February 2020.

[3] Durandt C, Potgieter J, Khoosal R, Nel J, Herd C, Mellet J, et al HIV and haematopoiesis. South African Medical Journal. 2019;109(8, Supplement 1):S41–S6. 10.7196/SAMJ.2019.v109i8b.13829 31662148

[4] Koka PS, Reddy ST. Cytopenias in HIV infection: mechanisms and alleviation of hematopoietic inhibition. Current HIV research. 2004;2(3):275–82. 15279591 10.2174/1570162043351282

[5] Opie J. Haematological complications of HIV infection. SAMJ: South African Medical Journal. 2012;102(6):465–8. 22668938 10.7196/samj.5595

[6] Pasha I. To Study the Hematological parameters as predictors of morbidity in patients with HIV infection. Global journal of medicine and public health.2004;3(1):1–11.

[7] Kathuria S, Bagga P, Malhotra S. Hematological manifestations in HIV infected patients and correlation with CD4 counts and anti retroviral therapy. J Contemp Med Res. 2016;3(12):3495–8.

[8] Vishnu P, Aboulafia DM. Haematological manifestations of human immune deficiency virus infection. British journal of haematology. 2015;171(5):695–709. 26452169 10.1111/bjh.13783

[9] Belperio PS, Rhew DC. Prevalence and outcomes of anemia in individuals with human immunodeficiency virus: a systematic review of the literature. The American journal of medicine. 2004;116(7):27–43. 10.1016/j.amjmed.2003.12.010 15050884

[10] O'Brien ME, Kupka R, Msamanga GI, Saathoff E, Hunter DJ, Fawzi WW. Anemia is an independent predictor of mortality and immunologic progression of disease among women with HIV in Tanzania. JAIDS Journal of Acquired Immune Deficiency Syndromes. 2005;40(2):219–25. 16186741 10.1097/01.qai.0000166374.16222.a2

[11] Volberding PA, Levine AM, Dieterich D, Mildvan D, Mitsuyasu R, Saag M, et al Anemia in HIV infection: clinical impact and evidence-based management strategies. Clinical infectious diseases. 2004;38(10):1454–63. 15156485 10.1086/383031

[12] Woldeamanuel GG, Wondimu DH. Prevalence of anemia before and after initiation of antiretroviral therapy among HIV infected patients at black lion specialized hospital, Addis Ababa, Ethiopia: a cross sectional study. BMC hematology. 2018;18(1):7. 29568529 10.1186/s12878-018-0099-y PMC5856395

[13] Jin Y, Li Q, Meng X, Xu Q, Yuan J, Li Z, et al Prevalence of anaemia among HIV patients in rural China during the HAART era. International journal of STD & AIDS. 2017;28(1):63–8. 26672003 10.1177/0956462415622866

[14] Harris RJ, Sterne JA, Abgrall S, Dabis F, Reiss P, Saag M, et al Prognostic importance of anaemia in HIV-1 infected patients starting antiretroviral therapy: collaborative analysis of prospective cohort studies in industrialized countries. Antiviral therapy. 2008;13(8):959. 19195321 PMC4507810

[15] Enawgaw B, Alem M, Addis Z, Melku M. Determination of hematological and immunological parameters among HIV positive patients taking highly active antiretroviral treatment and treatment naïve in the antiretroviral therapy clinic of Gondar University Hospital, Gondar, Northwest Ethiopia: a comparative cross-sectional study. BMC hematology. 2014;14(1):8. 24666771 10.1186/2052-1839-14-8 PMC3994311

[16] Melese H, Wassie MM, Woldie H, Tadesse A, Mesfin N. Anemia among adult HIV patients in Ethiopia: a hospital-based cross-sectional study. HIV/AIDS (Auckland, NZ). 2017;9:25. 10.2147/HIV.S121021 PMC5317259 28243151

[17] Deressa T, Damtie D, Workineh M, Genetu M, Melku M. Anemia and thrombocytopenia in the cohort of HIV-infected adults in northwest Ethiopia: a facility-based cross-sectional study. Ejifcc. 2018;29(1):36. 29765285 PMC5949617

[18] Gebremedhin KB, Haye TB. Factors Associated with Anemia among People Living with HIV/AIDS Taking ART in Ethiopia. Advances in hematology. 2019;2019. 10.1155/2019/9614205 PMC6421011 30941180

[19] Fekene TE, Juhar LH, Mengesha CH, Worku DK. Prevalence of cytopenias in both HAART and HAART naïve HIV infected adult patients in Ethiopia: a cross sectional study. BMC hematology. 2018;18(1):8. 29632668 10.1186/s12878-018-0102-7 PMC5887186

[20] Evans RH, Scadden DT. Haematological aspects of HIV infection. Best Practice & Research Clinical Haematology. 2000;13(2):215–30. 10942622 10.1053/beha.1999.0069

[21] Calenda V, Chermann JC. The effects of HIV on hematopoiesis. European journal of haematology. 1992;48(4):181–6. 1592096 10.1111/j.1600-0609.1992.tb01582.x

[22] Shi X, Sims MD, Hanna MM, Xie M, Gulick PG, Zheng Y-H, et al Neutropenia during HIV infection: adverse consequences and remedies. International reviews of immunology. 2014;33(6):511–36. 24654626 10.3109/08830185.2014.893301 PMC4873957

[23] Leroi C, Balestre E, Messou E, Minga A, Sawadogo A, Drabo J, et al Incidence of severe neutropenia in HIV-infected people starting antiretroviral therapy in West Africa. PloS one. 2017;12(1). 10.1371/journal.pone.0170753 PMC5266303 28122041

[24] Torre D, Pugliese A. Platelets and HIV-1 infection: old and new aspects. Current HIV research. 2008;6(5):411–8. 18855651 10.2174/157016208785861140

[25] Nassiri R. Avoiding antiretroviral-associated cytopenias. The Journal of the American Osteopathic Association. 2006;106(3):111–2. 16585370

[26] WHO. Haemoglobin concentrations for the diagnosis of anaemia and assessment of severity. World Health Organization; 2011.

[27] Tamir Z, Seid A, Haileslassie H. Magnitude and associated factors of cytopenias among antiretroviral therapy naïve Human Immunodeficiency Virus infected adults in Dessie, Northeast Ethiopia. PloS one. 2019;14(2). 10.1371/journal.pone.0211708 PMC6373930 30759131

[28] Gunda DW, Godfrey KG, Kilonzo SB, Mpondo BC. Cytopenias among ART-naive patients with advanced HIV disease on enrolment to care and treatment services at a tertiary hospital in Tanzania: A crosssectional study. Malawi Medical Journal. 2017;29(1):43–52. 28567196 10.4314/mmj.v29i1.9 PMC5442491

[29] Fan L, Li C, Zhao H. Prevalence and Risk Factors of Cytopenia in HIV-Infected Patients before and after the Initiation of HAART. BioMed Research International. 2020;2020. 10.1155/2020/3132589 PMC7008269 32090076

[30] Denue BA, Kida IM, Hammagabdo A, Dayar A, Sahabi MA. Prevalence of anemia and immunological markers in HIV-infected patients on highly active antiretroviral therapy in Northeastern Nigeria. Infectious Diseases: Research and Treatment. 2013;6:IDRT. S10477. 10.4137/IDRT.S10477 PMC3988622 24847174

[31] Sale A, Obi OS, Thomus JM, Waziri G, Bukar A, Erameh TO, et al Prevalence of Pancytopenia among Human Immuno-Virus (HIV) Sero-positive Clients attending Anti-retroviral Therapy (ART) Clinic in General Hospital Kachia, Kaduna State. Nigerian Biomedical Science Journal.2018;15(4):18–22.

[32] Ako S, Njunda L, Akum E, Benjamin P, Assob J. Hematological Related Disorders and Transfusion of HIV Patients on Highly Active Antiretroviral Therapy (HAART) in the South West Region of Cameroon: Hematological Monitory Parameters for HIV Follow-Up. J HIV Retrovirus. 2018;4(1):5.

[33] Omoregie R, Omokaro E, Palmer O, Ogefere H, Egbeobauwaye A, Adegue J, et al Prevalence of anaemia among HIV-infected patients in Benin City, Nigeria. Tanzania Journal of Health Research. 2009;11(1). 10.4314/thrb.v11i1.43242 19445097

[34] De Santis GC, Brunetta DM, Vilar FC, Brandao RA, de Albernaz Muniz RZ, de Lima GMN, et al Hematological abnormalities in HIV-infected patients. International Journal of Infectious Diseases. 2011;15(12):e808–e11. 21880530 10.1016/j.ijid.2011.08.001

[35] Assefa M, Abegaz WE, Shewamare A, Medhin G, Belay M. Prevalence and correlates of anemia among HIV infected patients on highly active anti-retroviral therapy at Zewditu Memorial Hospital, Ethiopia. BMC hematology. 2015;15(1):6. 26045966 10.1186/s12878-015-0024-6 PMC4455710

[36] Ageru TA, Koyra MM, Gidebo KD, Abiso TL. Anemia and its associated factors among adult people living with human immunodeficiency virus at Wolaita Sodo University teaching referral hospital. PloS one. 2019;14(10). 10.1371/journal.pone.0221853 PMC6785157 31596865

[37] Zerihun KW, Bikis GA, Muhammad EA. Prevalence and associated factors of anemia among adult human immune deficiency virus positive patients on anti-retroviral therapy at Debre tabor Hospital, Northwest Ethiopia. BMC research notes. 2019;12(1):168. 30909968 10.1186/s13104-019-4214-3 PMC6434868

[38] Kyeyune R, Saathoff E, Ezeamama AE, Löscher T, Fawzi W, Guwatudde D. Prevalence and correlates of cytopenias in HIV-infected adults initiating highly active antiretroviral therapy in Uganda. BMC infectious diseases. 2014;14(1):496. 25209550 10.1186/1471-2334-14-496 PMC4165997

[39] Egata G, Dereje B, Dessalegn G, Birhanu S. Prevalence of Anemia and associated factors among PHIVs attendants antiretroviral therapy clinics in public health institutions in Dire Dawa town, East Ethiopia. Journal of Medicine, Physiology and Biophysics. 2016;22.

[40] Petraro P, Duggan C, Spiegelman D, Hertzmark E, Makubi A, Chalamilla G, et al Determinants of anemia among human immunodeficiency virus-positive adults at care and treatment clinics in dar es salaam, Tanzania. The American journal of tropical medicine and hygiene. 2016;94(2):384–92. 26666698 10.4269/ajtmh.15-0587 PMC4751946

[41] Kumar MB, Thippeswamy T, Shankar R, Prathima C. Hematological abnormalities in early and advanced HIV infection patients. International journal of scientific study. 2016;3(11):1–5.

[42] Afari S, Blay E. Prevalence of Haematological and Serum Biochemical Abnormalities in HIV Infected Patients in Ghana, before and after Antiretroviral Therapy. Int J Virol AIDS. 2018;5(1):039.

[43] Mitra J, Horo SM. Analysis of haematological profile in HIV positive patients before and after antiretroviral therapy. Int J Health Sci Res. 2015;5(11):18–24.

[44] Taremwa IM, Muyindike WR, Muwanguzi E, Boum Y. Prevalence of HIV-related thrombocytopenia among clients at Mbarara Regional Referral Hospital, Mbarara, southwestern Uganda. Journal of blood medicine. 2015;6:109. 25926763 10.2147/JBM.S80857 PMC4403596

[45] Woldeamanuel GG, Wondimu DH. Prevalence of thrombocytopenia before and after initiation of HAART among HIV infected patients at black lion specialized hospital, Addis Ababa, Ethiopia: a cross sectional study. BMC hematology. 2018;18(1):9. 29760930 10.1186/s12878-018-0103-6 PMC5944097

[46] Nka AD, Sosso SM, Fokam J, Bouba Y, Teto G, Rachel RS, et al Thrombocytopenia according to antiretroviral drug combinations, viremia and CD4 lymphocytes among HIV-infected patients in Cameroon: a snapshot from the City of Yaoundé. BMC research notes. 2019;12(1):632. 31554515 10.1186/s13104-019-4664-7 PMC6761711

[47] Firnhaber C, Smeaton L, Saukila N, Flanigan T, Gangakhedkar R, Kumwenda J, et al Comparisons of anemia, thrombocytopenia, and neutropenia at initiation of HIV antiretroviral therapy in Africa, Asia, and the Americas. International Journal of Infectious Diseases. 2010;14(12):e1088–e92. 20961784 10.1016/j.ijid.2010.08.002 PMC3021118

[48] Santiago-Rodríguez EJ, Mayor AM, Fernández-Santos DM, Hunter-Mellado RF. Profile of HIV-infected hispanics with pancytopenia. International journal of environmental research and public health. 2016;13(1):38. 10.3390/ijerph13010038 PMC4730429 26703689

