# Supplementary material for: Prevalence of cytopenia and its associated factors among HIV infected adults on highly active antiretroviral therapy at Mehal Meda Hospital, North Shewa Zone, Ethiopia
Source: PLoS One. 2020 Sep 15;15(9):e0239215. doi: 10.1371/journal.pone.0239215 (PMC7491728; doi:10.1371/journal.pone.0239215)
Supplement: Attachment — Submitted filename: Response to Reviewers.docx [file pone.0239215.s003.docx]

Angesom Gebreweld

Mekelle University

Mekelle, Ethiopia

Email: [afsaha@gmail.com](mailto:afsaha@gmail.com)

**Date:** August 14, 2020

**To: PLOS ONE Journal Editorial Office**

**Dear Editorial:**

We are glad to write this response that our paper entitled **“**prevalence of cytopenia and its associated factors among HIV infected adults on highly active antiretroviral therapy at Mehal Meda Hospital, North Shewa Zone, Ethiopia, PONE-D-20-04588R1**”** has been accepted for publication in **PLOS ONE**. We are pleased to have an opportunity to make our paper revised and we have greatly appreciated the reviewers’ comments, and suggestions were very helpful overall. In revising the paper, we have carefully considered reviewers’ comments and suggestions on our revised submission. As instructed, we have attempted to succinctly explain changes made in reaction to all comments and reply to each comment in point-by-point fashion as follows:

**Response to reviewer**

We appreciate the reviewer’s comments. The followings are our point-by-point responses:

**Comment:** The authors have addressed most of my concerns. Although substantially improved from the first draft, the manuscript still requires additional thorough proof-reading and correction of grammatical/typographic errors such as consistency in verb tense, non-standard capitalization (e.g. "Male" instead of "male" in the middle of a sentence see line 256 as an example), missing words (e.g. see line 232 "Anemia *was* also...").

**Response:** We have corrected the grammatical/typographic errors, non-standard capitalizations, and missing words in the manuscript.

**Comment:** Additionally, the tables also require some revisions. One, each table should be able to stand alone and not rely on the text, hence the definitions of severity should be included in the footnote. Secondly, the standard number of decimal places for effect estimates (e.g. PR) is 2. In the tables the number of decimal places is inconsistent (sometimes 2 and sometimes 3). Additionally, there should be a space between the n and the %, e.g. 128 (66.0) instead of 128(66.0).

.

**Response:** We have included the definitions of severity in the footnote of Table 3.We have corrected the number of decimal places for PR in the tables and texts and we have put a space between n and % in the tables.

**Looking forward to hearing from you. Thank you again for your consideration!**

**Sincerely,**

**Angesom Gebreweld (BSc, MSc)**
